# Supplementary material for: Complete Plastome of Physalis angulata var. villosa, Gene Organization, Comparative Genomics and Phylogenetic Relationships among Solanaceae
Source: Genes (Basel). 2022 Dec 5;13(12):2291. doi: 10.3390/genes13122291 (PMC9778145; doi:10.3390/genes13122291)
Supplement: Supplementary file 1 [file genes-13-02291-s001.zip › Table S1.pdf]

Table S1 The 80 species belonging to 23 genera of Solanaceae, and four species used as outgroups

Number	Subfamily	Tribe	Genus	Species	Accession number	Groups
1	Solanoideae	Physaleae	*Acnistus*	*Acnistus arborescens*	KU568472	I-1
2			*Dunalia*	*Dunalia brachyacantha*	KP308151	I-1
3				*Dunalia solanacea*	KP998157	I-1
4				*Dunalia obovata*	KP280057	I-1
5			*Eriolarynx*	*Eriolarynx fasciculata*	KU306938	I-1
6			*Iochroma*	*Iochroma ellipticum*	KU323367	I-1
7				*Iochroma salpoanum*	KU315119	I-1
8				*Iochroma australe*	KU311000	I-1
9				*Iochroma stenanthum*	KP262399	I-1
10				*Iochroma tingoanum*	KP280175	I-1
11				*Iochroma loxense*	KP296185	I-1
12				*Iochroma nitidum*	KP294386	I-1
13				*Iochroma umbellatum*	KU310542	I-1
14				*Iochroma cyaneum*	KU319588	I-1
15				*Iochroma lehmannii*	KU310654	I-1
16			*Saracha*	*Saracha punctata*	KP280050	I-1
17			*Vassobia*	*Vassobia dichotoma*	KP294521	I-1
18			*Withania*	*Withania riebeckii*	BK010849	I-2
19				*Withania adpressa*	BK010847	I-2
20				*Withania coagulans*	MN216390	I-2
21			*Physalis*	*Physalis angulata* var. *villosa*	OM257167	I-3
22				*Physalis alkekengi* var. *franchetii* (Synonyms for *Alkekengi officinarum*)	MH045575	I-3
23				*Physalis chenopodifolia*	MN508249	I-3

24			*Physalis philadelphica*	MN192191	I-3
25			*Physalis minima*	MH045577	I-3
26			*Physalis pubescens*	MH045576	I-3
27			*Physalis angulata*	MH045574	I-3
28			*Physalis pruinosa*	MH019243	I-3
29			*Physalis peruviana*	MH019242	I-3
30		*Tubocapsicum*	*Tubocapsicum anomalum*	MW829600	III
31	Capsiceae	*Capsicum*	*Capsicum lycianthoides*	KP274856	II
32			*Capsicum frutescens*	KR078312	II
33			*Capsicum annuum* var. *glabriusculum*	KR078311	II
34			*Capsicum annuum*	JX270811	II
35			*Capsicum eximium*	KX913220	II
36			*Capsicum tovarii*	KX913219	II
37			*Capsicum chacoense*	KX913218	II
38			*Capsicum chinense*	KX913217	II
39			*Capsicum galapagoense*	KX913216	II
40			*Capsicum baccatum* var *baccatum*	KR078314	II
41	Solaneae	*Solanum*	*Solanum habrochaites*	KP117023	III
42			*Solanum chilense*	KP117021	III
43			*Solanum pennellii*	HG975452	III
44			*Solanum nigrum*	KM489055	III
45			*Solanum bulbocastanum*	DQ347958	III
46			*Solanum cheesmaniae*	KP117020	III
47			*Solanum pimpinellifolium*	KP117027	III
48			*Solanum commersonii*	KM489054	III
49			*Solanum tuberosum*	DQ231562	III

50				*Solanum neorickii*	KP117025	III
51				*Solanum lycopersicum*	KP117024	III
52				*Solanum peruvianum*	KP117026	III
53				*Solanum galapagense*	KP117022	III
54		Datureae	*Datura*	*Datura stramonium*	NC_018117	IV
55			*Trompettia*	*Trompettia cardenasianum*	KU310932	IV
56		Lycieae	*Lycium*	*Lycium chinense*	MN102357	V-1
57				*Lycium ferocissimum*	MN866909	V-1
58				*Lycium ruthenicum*	MG729825	V-1
59				*Lycium barbarum*	MG729823	V-1
60		Hyoscyameae	*Anisodus*	*Anisodus tanguticus*	MK347419	V-2
61				*Anisodus acutangulus*	MN781973	V-2
62			*Atropa*	*Atropa belladonna*	NC_004561	V-2
63			*Atropanthe*	*Atropanthe sinensis*	MK411818	V-2
64			*Hyoscyamus*	*Hyoscyamus niger*	KF248009	V-2
65			*Physochlaina*	*Physochlaina orientalis*	MK492324	V-2
66				*Physochlaina physaloides*	MN262642	V-2
67			*Przewalskia*	*Przewalskia tangutica*	KY352315	V-2
68			*Scopolia*	*Scopolia parviflora*	KU900232	V-2
69	Nicotianoideae	Nicotianeae	*Nicotiana*	*Nicotiana tabacum*	Z00044	VI
70				*Nicotiana attenuata*	MG182422	VI
71				*Nicotiana otophora*	KU051626	VI
72				*Nicotiana undulata*	JN563929	VI
73				*Nicotiana sylvestris*	NC_007500	VI
74				*Nicotiana glauca*	MT985321	VI
75				*Nicotiana debneyi*	MT985319	VI

76			*Nicotiana repanda*	MT985323	VI
77			*Nicotiana stocktonii*	MT985322	VI
78			*Nicotiana suaveolens*	MT985320	VI
79	Petunioideae	*Petunia*	*Petunia exserta*	MT644125	VII
80			*Petunia x hybrida*	MF459662	VII
81		*Bacopa*	*Bacopa monnieri*	MN736955	Outgroup
82		*Digitalis*	*Digitalis lanata*	KY085895	Outgroup
83		*Rehmannia*	*Rehmannia glutinosa*	MG977439	Outgroup
84		*Scrophularia*	*Scrophularia buergeriana*	KP718626	Outgroup
