# Supplementary material for: Complete Plastome of Physalis angulata var. villosa, Gene Organization, Comparative Genomics and Phylogenetic Relationships among Solanaceae
Source: Genes (Basel). 2022 Dec 5;13(12):2291. doi: 10.3390/genes13122291 (PMC9778145; doi:10.3390/genes13122291)
Supplement: Supplementary file 1 [file genes-13-02291-s001.zip › Table S2.pdf]

Table S2. Predicted RNA editing site in the *P. angulata* var. *villosa* plastome.

Gene	Nucleotide Position	Amino Acid Position	Codon Conversion	Amino Acid Conversion	Score
atpA	383	128	ACC=> ATC	T => I	1.00
	424	142	CAC=> TAC	H => Y	1.00
	772	258	CAT => TAT	H => Y	1.00
	868	290	CAT => TAT	H => Y	1.00
	877	293	CCT => TCT	P => S	1.00
	1142	381	TCG => TTG	S => L	1.00
atpB	322	108	CTT=> TTT	L => F	1.00
	892	298	CAC => TAC	H => Y	1.00
	968	323	ACC => ATC	T => I	1.00
	1159	387	CAC => TAC	H => Y	1.00
	1190	397	CCC => CTC	P => L	1.00
atpF	430	144	CCA => TCA	P => S	0.86
	508	170	CTC => TTC	L=> F	1.00
	596	199	TCG => TTG	S => L	1.00
	1151	384	CCA => CTA	P => L	1.00
atpI	74	25	ACT => ATT	T => I	1.00
	170	57	CCC => CTC	P => L	1.00
	371	124	CCC => CTC	P => L	1.00
	463	155	CCA => TCA	P => S	1.00
	499	167	CAC => TAC	H => Y	1.00
	568	190	CCG => TCG	P => S	1.00
	590	197	ACC => ATC	T => I	1.00
	650	217	ACC => ATC	T => I	1.00
matK	152	51	GCC => GTC	A => V	0.83
	568	190	CCT => TCT	P => S	0.83
	685	229	CCT => TTT	P => F	0.86
	686	229	CCT => TTT	P => F	0.86
	1246	416	CAT => TAT	H => Y	1.00
	1274	425	CCC => CTC	P => L	1.00
ndhA	344	115	ACC => ATC	T => I	1.00
	760	254	CTT => TTT	L => F	1.00
	842	281	GCT => GTT	A => V	1.00
	940	314	CTC => TTC	L => F	1.00
	1988	663	TCA => TTA	S => L	1.00
	2003	668	TCC => TTC	S => F	1.00
ndhB	149	50	TCA => TTA	S => L	1.00
	467	156	CCA => CTA	P => L	1.00
	586	196	CAT => TAT	H => Y	1.00
	611	204	TCA => TTA	S => L	0.80
	737	246	CCA => CTA	P => L	1.00
	746	249	TCT => TTT	S => F	1.00
	967	323	CAT => TAT	H => Y	1.00
	1462	488	CAC => TAC	H => Y	1.00
	1759	587	CAT => TAT	H => Y	1.00
	1891	631	CCT => TTT	P => F	1.00
	1892	631	CCT => TTT	P => F	1.00
	2072	691	TCA => TTA	S => L	1.00
	2204	735	CCT => CTT	P => L	1.00
ndhD	83	28	ACC=> ATC	T => I	0.80

	668	223	CCA => CTA	P => L	0.80
	716	239	TCG => TTG	S => L	1.00
	983	328	ACC => ATC	T => I	1.00
	1082	361	CCA => CTA	P => L	1.00
	1124	375	TCC => TTC	S => F	1.00
	1486	496	CCA => TCA	P => S	1.00
ndhF	134	45	CCC => CTC	P=> L	1.00
	151	51	CCC => TTC	P=> F	1.00
	152	51	CCC => TTC	P =>F	1.00
	164	55	TCG => TTG	S => L	1.00
	526	176	CTC => TTC	L => F	1.00
	917	306	CCA => CTA	P => L	1.00
	1079	360	ACC => ATC	T => I	1.00
	1478	493	CCA => CTA	P => L	1.00
	1754	585	ACA => ATA	T => I	1.00
	1759	587	CCC => TCC	P => S	1.00
ndhG	157	53	CCA => TCA	P => S	1.00
petB	1364	455	CCA=> CTA	P => L	1.00
psaB	80	27	TCC => TTC	S => F	1.00
	349	117	CCA => TCA	P => S	1.00
	401	134	CCA=> CTA	P => L	1.00
	418	140	CCA => TCA	P => S	0.86
	737	246	TCG => TTG	S => L	1.00
	821	274	CCA => CTA	P => L	0.86
	977	326	TCT => TTT	S => F	1.00
	1202	401	GCC => GTC	A => V	1.00
	1258	420	CCC=> TCC	P => S	1.00
	1319	440	CCC => CTC	P => L	1.00
	1544	515	CCC => CTC	P => L	1.00
	1631	544	CCC => CTC	P => L	1.00
	1822	608	CCC => TTC	P => F	1.00
	1823	608	CCC=> TTC	P => F	1.00
	1834	612	CCA => TCA	P => S	1.00
	1889	630	TCG=> TTG	S => L	1.00
	1981	661	CTC => TTC	L => F	1.00
	2027	676	CCA=> CTA	P => L	1.00
psbE	200	67	TCG => TTG	S => L	1.00
psbF	19	7	CAT => TAT	H=>Y	1.00
rpl2	199	67	CTT => TTT	L => F	1.00
	536	179	ACA => ATA	T => I	1.00
	841	281	CCT => TCT	P => S	1.00
rpl20	415	139	CCG => TCG	P => S	1.00
rpl23	58	20	CAT => TAT	H => Y	1.00
	230	77	CCC => CTC	P => L	1.00
rpoA	461	154	ACT => ATT	T => I	1.00
	602	201	ACT => ATT	T => I	0.86
	866	289	TCG => TTG	S => L	1.00
	965	322	ACA => ATA	T => I	0.86
rpoB	223	75	CCC => TCC	P => S	1.00
	263	88	CCG => CTG	P => L	0.86
	491	164	ACT => ATT	T => I	1.00
	680	227	TCC => TTC	S => F	1.00
	1118	373	GCT => GTT	A => V	0.86

	1330	444	CCG => TCG	P => S	1.00
	1486	496	CCC => TTC	P => F	1.00
	1487	496	CCC => TTC	P => F	1.00
	2312	771	ACC => ATC	T => I	1.00
	2510	837	GCC => GTC	A => V	1.00
	2692	898	CCT => TCT	P => S	1.00
	2822	941	ACC => ATC	T => I	0.86
	3004	1002	CAC => TAC	H => Y	0.86
	3140	1047	CCT => CTT	P => L	1.00
rpoC1	953	318	TCC => TTC	S => F	1.00
	1280	427	TCG=> TTG	S => L	1.00
	1571	524	TCG => TTG	S => L	1.00
	1642	548	CCC=> TCC	P => S	1.00
	1727	576	ACT=> ATT	T => I	1.00
	1760	587	CCT => CTT	P => L	1.00
	1811	604	TCT=> TTT	S => F	1.00
	2282	761	CCA=> CTA	P => L	1.00
	2452	818	CAT => TAT	H => Y	0.86
	2522	841	TCA => TTA	S => L	1.00
	2684	895	ACT=> ATT	T => I	0.86
rpoC2	341	114	TCT => TTT	S => F	1.00
	721	241	CAC => TAC	H => Y	1.00
	863	288	TCG => TTG	S => L	1.00
	868	290	CAC => TAC	H => Y	1.00
	982	328	CTC=> TTC	L => F	1.00
	1312	438	CCC => TCC	P => S	0.86
	1562	521	TCT => TTT	S => F	1.00
	2306	769	TCT => TTT	S => F	1.00
	2728	910	CCC => TCC	P => S	1.00
	2743	915	CCA=> TCA	P => S	0.86
	2858	953	TCG => TTG	S => L	1.00
rps2	310	104	CGG=> TGG	R => W	1.00
	443	148	CCT=> CTT	P => L	1.00
	512	171	CCT => CTT	P => L	1.00
	631	211	CCA => TCA	P => S	1.00
	644	215	ACC=> ATC	T => I	1.00
rps8	14	5	ACA=> ATA	T => I	1.00
	113	38	TCG => TTG	S => L	1.00
	332	111	TCG => TTG	S => L	1.00
	340	114	CCT=> TCT	P => S	1.00
	398	133	CCT => CTT	P => L	1.00
	403	135	CAT=> TAT	H => Y	1.00
rps14	230	77	TCG => TTG	S => L	1.00
	782	261	CCA => CTA	P => L	0.83
	821	274	TCT => TTT	S => F	1.00
ycf3	232	78	CTT=> TTT	L => F	1.00
	481	161	CAT => TAT	H => Y	1.00
	628	210	CAC => TAC	H => Y	1.00
