# Supplementary figures and images for: Complete Plastome of Physalis angulata var. villosa, Gene Organization, Comparative Genomics and Phylogenetic Relationships among Solanaceae
Source: Genes (Basel). 2022 Dec 5;13(12):2291. doi: 10.3390/genes13122291 (PMC9778145; doi:10.3390/genes13122291)

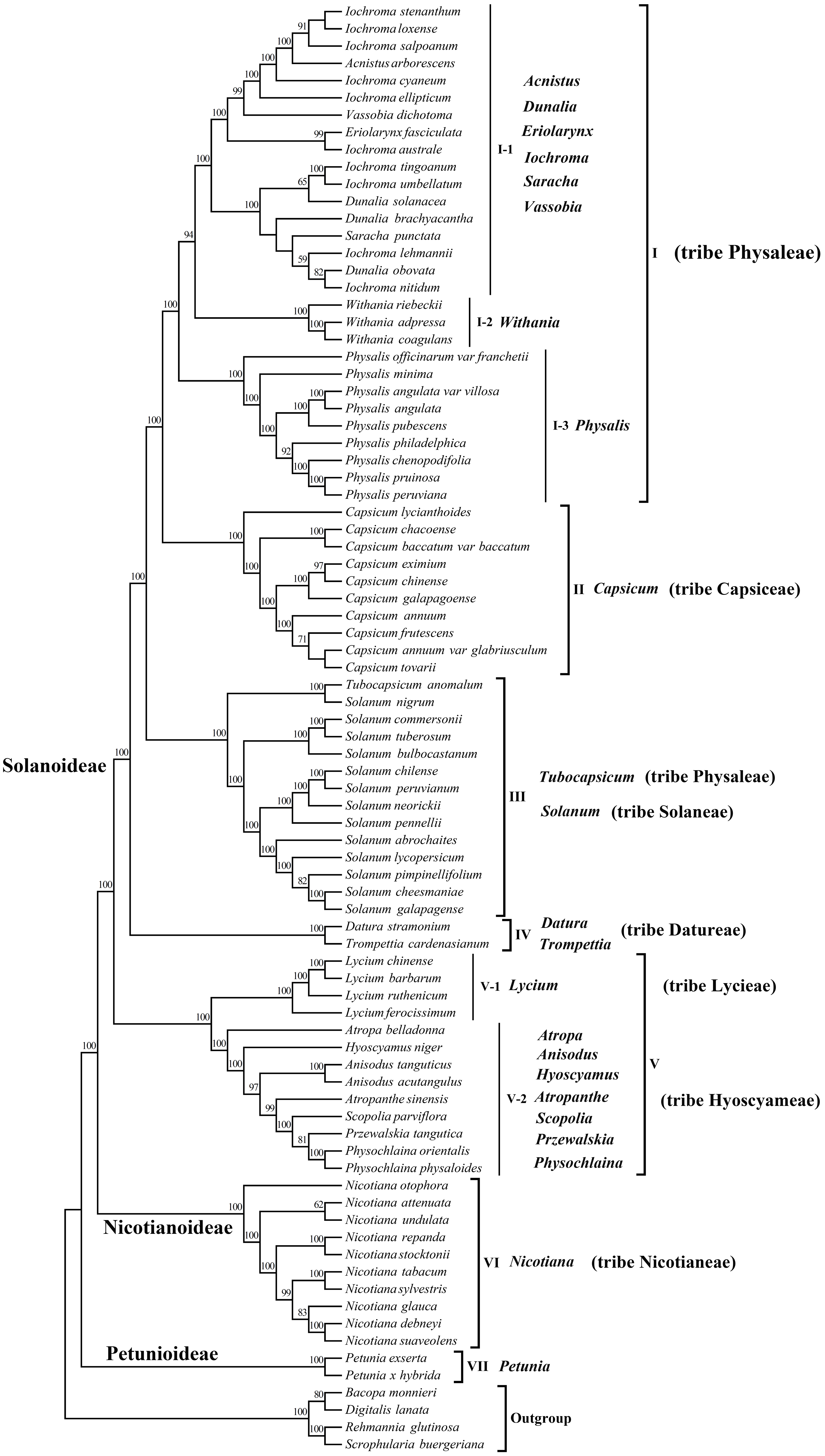

Supplement: Supplementary file 1 [file genes-13-02291-s001.zip › Figure S1.tif]
